# Bamboo–PCM: Comparative Analysis of Phase Change Material-Impregnated *Dendrocalamus giganteus* Culm Behavior Exposed to Thermal Variation in Wind Tunnel Assay

**DOI:** 10.3390/ma18030675

**Published:** 2025-02-03

**Authors:** Fabrício Rezende Fontenelle, Túlio Caetano Guimarães, Tadayuki Yanagi Junior, Marcelo Bahuti, Romildo Dias Toledo Filho, Eddie Koenders, Saulo Rocha Ferreira

**Affiliations:** 1 Department of Design, Representation, Technology of Architecture and Urban Planning, Federal University of Juiz de Fora, P.O. Box 20010, Juiz de Fora 36016-970, MG, Brazil; fabricio.fontenelle@ufjf.br; 2 Department of Forest Sciences, Federal University of Lavras, P.O. Box 3037, Lavras 37200-900, MG, Brazil; tuliocaetano@hotmail.com (T.C.G.); yanagi@ufla.br (T.Y.J.); marcelo_bahuti@hotmail.com (M.B.); 3 Department of Geosciences and Natural Resource Management, University of Copenhagen, Rolighedsvej 23, 1958 Frederiksberg C, Denmark; 4 Department of Civil Engineering, Federal University of Rio de Janeiro, P.O. Box 68506, Rio de Janeiro 21941-972, RJ, Brazil; toledo@coc.ufrj.br; 5 Institute of Construction and Building Materials, Technical University of Darmstadt, L5, 06 207, Franziska-Braun-Straße 3, 64287 Darmstadt, Germany; koenders@wib.tu-darmstadt.de; 6 Department of Engineering, Federal University of Lavras, P.O. Box 3037, Lavras 37200-900, MG, Brazil

**Keywords:** bamboo, phase change material, thermal energy storage, thermal characterization, wind tunnel, *Dendrocalamus giganteus*

## Abstract

The construction industry’s pursuit of eco-friendly materials has sparked interest in bamboo, a renewable resource with exceptional physical and mechanical properties. This study analyzed the integration of *Dendrocalamus giganteus* bamboo with phase change materials (PCMs) to enhance thermal energy storage in building applications, aiming to improve temperature regulation and reduce energy consumption for climate control. The study compared the performance of bamboo impregnated with an industrial PCM or coconut oil, used in conjunction with a polyurethane resin (PU) coating treatment, assessing their thermal regulation performance against traditional building materials such as ceramic tiles, fiber cement, and metal sheets. From an anatomical perspective, the pores within bamboo culms offered ample space for PCM storage, resulting in a substantial heat storage capacity. Thermal behavior tests conducted in a wind tunnel revealed that the impregnated bamboo samples effectively mitigate temperature fluctuations by aligning them with the PCM’s phase change temperature. Additionally, it was observed that air flow velocity had an impact on this phenomenon. The study concluded that bamboo culms impregnated with PCM hold promise for temperature regulation in construction applications, with variations in airflow exerting an impact on the outcomes obtained.

## 1. Introduction

Bamboo stands as a sustainable natural resource with substantial potential to substantially reduce the environmental impact of the construction industry. This versatile plant species seems tailor-made for use in structural applications. Its widespread availability in regions where it is cultivated, ease of distribution, rapid growth cycle, and favorable physical and mechanical properties make it an ideal material for construction. As an eco-friendly alternative to materials derived from non-renewable resources or native forests often exploited without proper management, bamboo offers a sustainable solution, particularly in areas where its cultivation is established and supported [1].

On a parallel front, innovative research aimed at enhancing materials’ thermal capacity and efficiency has made significant strides in exploring the capabilities of Phase Change Materials (PCMs). These materials possess the capacity to store latent energy, and when integrated into a building, they effectively stabilize temperature fluctuations, reducing the need for artificial climate control in response to natural climate variations [2]. Within these materials, during a phase change caused by temperature variations that prevent the PCM from remaining in its prior state, there is a reorganization of intermolecular chemical bonds. This transformation necessitates the transfer of energy in both endothermic and exothermic directions, depending on the direction of the transition [3]. PCMs, at their melting or evaporation points, solidification or condensation, continue to absorb or release heat without altering their temperature for a certain duration, leading to a latent heat accumulation during this state transition.

PCMs encompass a range of chemical substances, with the most common ones falling into the organic category, predominantly paraffins and fatty acids. Additionally, inorganic substances like metals and hydrated salts can be utilized, as well as eutectic PCMs [4]. For this study, a bio-based organic PCM with a phase change temperature of approximately 24 °C was chosen, in addition to coconut oil, which has also been studied for its thermal storage potential at similar temperatures [5].

Shape stabilization methods are often necessary prior to the application of PCMs due to potential leakage of solid–liquid PCMs when in their liquid state. Porous materials have exhibited intriguing capabilities among the various materials used for PCM containment [6]. In the vacuum impregnation method, liquid PCM is adsorbed into the pores of a porous structure under vacuum conditions, leading to a shape-stable composite material [7]. A vast number of porous materials have been studied for this purpose, such as silica [8], silicon dioxide [9], expanded vermiculite [10], porous carbon [11], and expanded graphite [12]. Recent research has also been looking into the possibility of leveraging the inherent qualities of naturally porous lignocellulosic materials, such as jute [13] , to utilize them as support materials for PCM. This research aimed to use bamboo as a PCM support material, exploring the potential of impregnating PCM into the voids of bamboo’s anatomical structure through a vacuum impregnation method to enhance its thermal efficiency.

The bamboo species examined in this study, *Dendrocalamus giganteus*, is abundantly found in Brazilian territory and is frequently employed for rustic and artisanal purposes in traditional communities [14]. Nevertheless, its industrial utilization or processing for commercially viable applications remains in its early stages. This research endeavors to unlock another potential advantage to utilizing *Dendrocalamus giganteus* bamboo by combining the materials’ inherent strengths with PCM. A prior study [15] has shown that bamboo has promising attributes for this purpose.

A wind tunnel is a controlled experimental setup designed to simulate the effects of air movement over objects, making it an ideal tool for studying how materials interact with airflow and temperature variations [16]. In the context of this research, the wind tunnel is used to assess the thermal performance of *Dendrocalamus giganteus* bamboo impregnated with PCM under varying wind speeds. This setup allows for useful measurements of heat exchange processes to evaluate the effectiveness of PCM-impregnated bamboo for thermal energy storage in building applications. The wind tunnel can simulate how airflow influences temperature regulation, a factor often overlooked in traditional static thermal analysis.

The primary objective of this research is to assess the impact of wind flow speed on the thermal performance of PCM-impregnated bamboo, comparing it with other commonly used building materials. The aim is to preserve bamboo’s inherent qualities while incorporating a sufficient quantity of PCM to bestow it with the desired thermal capacity for effective functionality as a thermal energy storage material.

## 2. Materials and Methods

**Figure 1:**
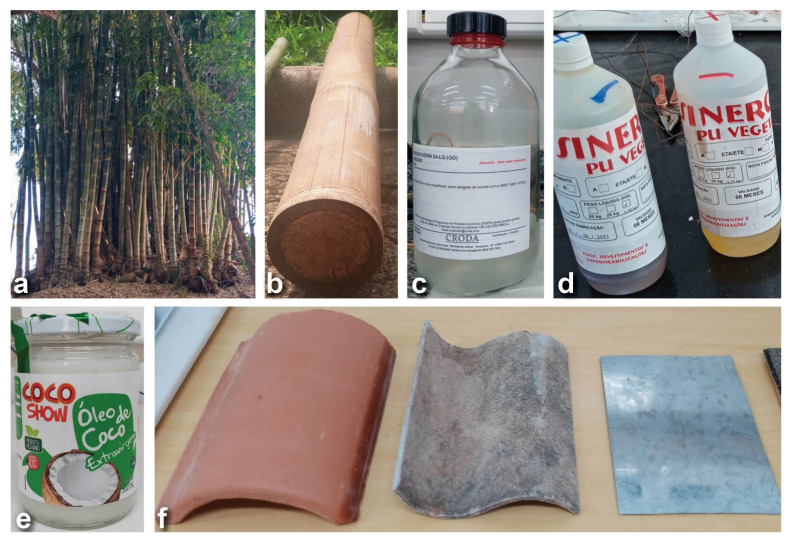
Materials: *Dendrocalamus giganteus* clump where the research material was harvested (**a**); piece of bamboo culm as used (**b**); commercial CrodaTherm 24 PCM (**c**); resin used for sealing, PU vegetal-type “V” commercial (**d**). Commercial coconut oil (**e**) and used specimens of building materials (**f**), respectively, from left to right: ceramic tiles, fiber cement, and metal sheet.

The primary material used in the various experimental stages was *Dendrocalamus giganteus* (D.) bamboo culms, commonly known as “Giant bamboo” in Brazil. These were harvested at coordinates of UTM Zone 23k: latitude: 7,622,850.81 mS and longitude: 545,461.37 mE. It is an abundant clumping exotic species in the Southeast region of Brazil, used in civil construction and for producing slats for laminates, with large dimensions. Figure 1a illustrates the clump from where the specimens were extracted and the kind of section of stalks that were selected (Figure 1b) and used for impregnation.

The phase change material (PCM) used was CrodaTherm 24 (Figure 1c), a bio-based organic PCM with a melting temperature of approximately 24 °C and a solidification temperature of approximately 21 °C. It was obtained through a donation from Croda International Plc (Campinas, Brazil) [17]. This PCM has a specific temperature range for phase change that is very close to the ideal thermal comfort temperature for use in buildings. The physical properties of this PCM can be found in Table 1 [13]. Extra virgin culinary coconut oil, Cocoshow brand from COPRA (Maceió, Brazil) (Figure 1e), purchased from a local supermarket, was also used as an alternative PCM. Its solidification temperature is 25 °C and the reported composition on the label is as follows in terms of fatty acids (g/100 g): C6:0 Caproic: 0.48, C8:0 Caprylic: 6.61, C10:0 Capric: 5.22, C12:0 Lauric: 44.29, C14:0 Myristic: 17.68, C16:0 Palmitic: 8.40, C18:0 Stearic: 3.00, C18:1 Omega 9 oleic: 5.78, and C18:2 Omega 6 Linoleic: 0.93.

Additionally, a PU Vegetal-Type “V” sealing resin (PU Vegetal Sinergia, Araraquara, Brazil) (Figure 1d), a two-component polyurethane waterproofing agent of vegetable origin (derived from castor oil—*Ricinus Communis*) [18], was used for coating. This resin has application and performance characteristics suitable for the sealing, durability, and waterproofing requirements of the research. Its natural origin from a renewable plant resource adds to its appeal. The resin presents an average density of 1.02 g/cm³, a mechanical strength at a compressive stress of 26 MPa, a Young Modulus of 1.8 MPa, and a Shore D hardness of 55.

Three other commonly used materials in the construction industry for building envelopes were also tested: ceramic tiles, fiber cement, and metal sheet, as shown in Figure 1f.

The research methodology essentially involved the following processes: the collection and preparation of samples for analysis, the physical and morphological characterization of the collected bamboo, a thermal analysis with air flow to scrutinize the effect of impregnated PCM compared with other commonly used building materials, the macroscopic and microscopic visual examination of the samples, as well as chemical characterization.

### 2.1. Cutting and Preparation of Stalks

**Figure 2:**
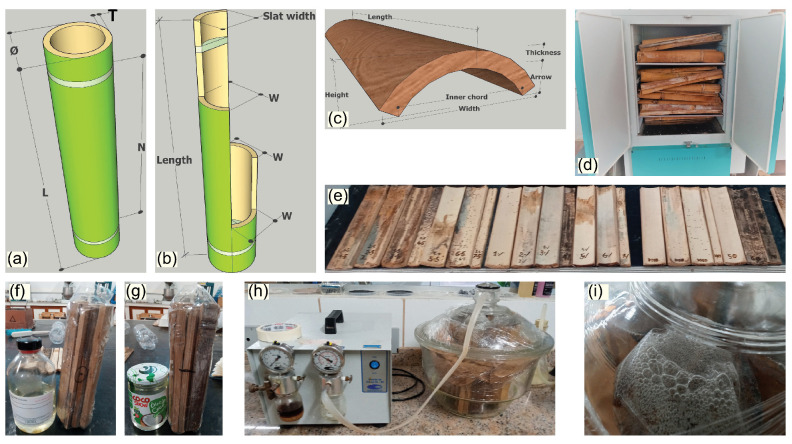
Original shape of the sample from the impregnation process (**a**); schematic of the longitudinal cut of the stalks (**b**); final slat shape (**c**); manufactured specimens (**e**); drying oven process (**d**); sample conditioning for impregnation (**f**,**g**); vacuum pump treatment (**h**); prominent bubbles on the top of samples in the impregnation process (**i**).

The cutting and preparation of stalks and production of assay specimens followed a procedure described in detail in a previous work [15]. From the collected bamboo samples, sections of the middle third of the bamboo stalk were extracted, in accordance with [19]. The bamboo stalks were subjected to a two-week period of natural drying or curing, after which they were longitudinally cut to the appropriate dimension of 250 mm as shown in Figure 2a. The prepared specimens were then placed in an oven for induced drying (Figure 2d), following established procedures documented in the literature [20,21,22].

### 2.2. PCM Impregnation and Coating of Specimens

The treatments applied to the bamboo culms are summarized in Table 2. Three treatments were considered: PU coating, PCM impregnation followed by PU coating, and coconut oil impregnation followed by PU coating. These treatments were selected to investigate not only the influence of PCM but also any potential effects, particularly concerning thermal behavior, associated with the impregnation of the coconut oil when exposed to variation in temperature and wind.

The PCM impregnation of the produced bamboo specimens employed a vacuum-assisted method, as described in [15]. The specimens were immersed in liquid PCM in a container inside a laboratory desiccator connected to a vacuum pump (Figure 2h). After completing the impregnation, excess PCM was removed and the specimens were stored at −6 °C.

The coating process was accomplished by mixing the PU resin components, but with a solvent dilution to enable the use of a compressed air spray gun. The test specimens were placed on a suitable support for easier application (Figure 2e).

### 2.3. Characterization

Morphological characteristics of bamboo samples from the *D. giganteus* species are shown in Table 3, including their average dimensions and characteristics of the nodes.

The samples were exposed to natural air drying to reach hygroscopic equilibrium [20,22,23], and the determination of apparent density was the ratio between the dry mass (weighed using a precision scale following oven drying) and the water-saturated volume (determined using the displaced liquid method based on Archimedes’ principle), while considering the irregular shape of the samples [20,21]. The mass measurements were also taken before and after impregnation, thus enabling the determination of the content of PCM or coconut oil in the final sample.

#### 2.3.1. Microscopy and Ultrastructural Analyses

The analysis of the microstructure of the bamboo species was carried out through several microscopy methods, which included light optical microscopy as well as the use of the following instruments: a Stereomicroscope with epifluorescence SMZ 1500 (Nikon), a Confocal Microscope LSM 780 (Carl Zeiss), and a Ultra-high-resolution Field Emission Scanning Electron Microscope-STEM-CLARA model (TESCAN, 2021). To observe internodal regions, and cross-sectional cuts to capture images of the stalks [24,25]. Samples measuring approximately 10 mm × 5 mm × 5 mm were derived from bamboo strips.

#### 2.3.2. Thermogravimetric Analysis (TGA)

Samples were processed into powder or fine grains for thermogravimetric analysis (TGA) and infrared spectroscopy. TGA was performed to evaluate the thermal stability of the specimens. A TGA Q500 TA Instruments thermal analyzer (New Castle, DE, USA) was used. Between 7 and 10 mg of powdered sample were heated from 25 to 1000 °C at 10 °C/min. The analysis atmosphere was synthetic air atmosphere (80% N_2_ and 20% O_2_) flowing at 60 mL/min.

#### 2.3.3. Fourier-Transform Infrared Spectroscopy (FTIR)

Fourier-transform infrared spectroscopy (FTIR) and vibrational infrared spectroscopy analyses were performed in a Varian 600-IR Fourier-transform infrared (FTIR) spectrometer (Agilent Technologie, Santa Clara, CA, USA) equipped with a GladiATR accessory (Pike Technologies, Fitchburg, WI, USA) for attenuated total reflectance (ATR) measurements at a 45° angle, utilizing a zinc selenide crystal. The spectral range analyzed was 400 to 4000 cm^−1^, with a resolution of 2 cm^−1^ and 32 scans.

### 2.4. Thermal Variation in Wind Tunnel Assay

**Figure 3:**
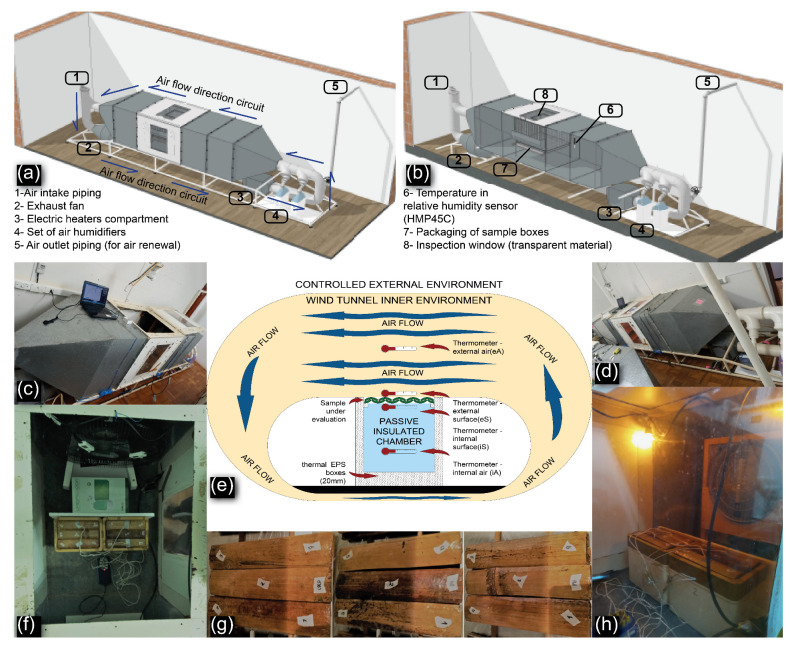
Schematic perspective (**a**) and section (**b**) of the wind tunnel equipment with the main accessories [26]; experimental apparatus (**c**,**d**) equipped with thermal insulating boxes (**f**), extra fans and monitoring thermometers (**h**); schematic diagram (**e**) of the setup environment with the location of the temperature sensors; groups of bamboo specimens from the three treatments (**g**).

The wind tunnel’s main feature is the ability to introduce airflow at various speeds into the set of parameters being evaluated. Another equipment designed for analyzing thermal variations in static air, referred to as passive chamber, was used in conjunction with the wind tunnel [15]. The experimental design consisted of evaluating the effect of different staggered wind speeds on the heat exchange processes occurring on the samples with a constant upward and downward temperature variation. In summary, the wind tunnel is a large circular circuit with inducing fans to create airflow, heating elements to control temperature, and equipment for regulating the relative humidity of the air within the system as shown in Figure 3.

The wind tunnel (Figure 3a–d) was equipped with a set of electrical resistances (totaling 2400 W), air humidifiers (total flow of 600 mL of water), an exhaust fan (40 cm in diameter and flow of 4200 m/h), and a lighting system. No flow straightener was used. In addition, inside the tunnels, a set of thermally insulated boxes was installed, performing the function of passive chambers (Figure 3e,f,h).

The passive chambers setup was based on a previous study [15]. It consisted of two 8-liter polystyrene (EPS) thermal insulation boxes with a 20 mm thickness placed side-by-side. These boxes were stacked with an EPS support, which introduced a septum between them and the external environment, serving as the placement area for the samples to be evaluated, effectively acting as a diaphragm which, due to its position, is also subject to wind action on its outer surface. The objective of the test was to assess the attenuation of thermal load between the two environments (wind tunnel air and passive chamber internal air) with initially equal temperatures.

In order to accelerate the temperature rise, the tunnel was also equipped with a heat-emitting element, in this case, an incandescent bulb (glass bulb with a Tungsten filament and E27 non-ferrous socket, with a power of 15 W and a voltage of 127 V). The insulated boxes (passive chambers) received the heat variation from the “external air”, which was attenuated by the samples (Figure 3e), creating an obstacle between the two environments, as outlined in the scheme presented in Table 4.

To ensure accurate temperature measurements, eight type K thermocouples (Model 21N029, HiKaru Company, São Paulo, Brazil), with a 3 mm probe and 24 AWG, were used in a data collection system, on the external (eS) and internal surfaces (iS) of the samples with temperature measurement and recording capabilities, boasting an accuracy of four decimal places in °C. Temperature readings were periodically recorded at predefined time intervals using software provided by the manufacturer of the commercial equipment, the Pico Technology USB TC-08 thermocouple data logger with PicoLog 6 data logging software . We also used, both in the tunnel environment, referred to as external air (eA), and in the internal environment of the passive chamber, referred to as internal air (iA), six HOBO (type onset 1-800-Loggers) temperature and relative humidity sensors, in addition to the sensor present in the tunnel type HMP45C (Vaisala^®^) with accuracies of 0.3 °C.

To conduct the experiment, it was important to note that the PCM used had a melting temperature of approximately 24 °C, and the heat storage process and the phenomenon under investigation occurred within this temperature range. Consequently, the experiment was planned to allow the temperature to traverse this range with a safety margin both above and below this value. This was necessary to observe the phenomenon in both its heating and cooling phases.

The experimental procedure commenced with the organization of the thermometers and parameters in the application to ensure accurate data recording (data collection frequency, designations, etc.). Subsequently, the wind tunnel heating system and the incandescent bulb was turned on, initiating the temperature increase phase in the system, referred to as Phase 1. The room where the experiment was conducted was climate-controlled at a temperature of approximately 19 °C, below the PCM’s melting point. Therefore, the wind tunnel setup needed to elevate the temperature above 24 °C but not significantly beyond 30 °C to avoid a substantial deviation from the ambient climate. This choice determined the power of the heating element. In Phase 1 (heating) of the experiment, the temperature in the wind tunnel was raised to values exceeding 35 °C, until a steady state was reached and temperatures stabilized, a process that took 120 min.

In Phase 2 (cooling), the wind tunnel, which contributed heat, was turned off. The air intake piping, air outlet piping, and the inspection window were opened. In addition, ice containers were placed inside the wind tunnel environment to accelerate the cooling of the system. This allowed a rapid temperature reduction in the setup temperature. Gradually, the influence of the barrier/sample on this cooling process was also noted. This temperature reduction and stabilization process also took 120 min.

The thermometers in the system were positioned at four levels (Figure 3e), with duplicates, to guarantee the reliability of the collected data. The frequency of temperature recording was every 5 s and they were arranged as follows:

- External Air (eA) Temperature sensors: positioned at the geometric center of the wind tunnel volume.
- External Surface of the specimen (eS) temperature sensors: placed on the surface exposed to the environment of the wind tunnel of the evaluated barrier.
- Internal Surface of the specimen (iS) temperature sensors: placed on the surface exposed to the environment of the passive chamber of the evaluated barrier.
- Internal Air (iA) temperature sensors: positioned at the geometric center of the volume of the passive chambers.

Throughout the experimental period, the setup was kept in a relative humidity of 60%, automatically controlled by the monitoring system of a datalogger (CR1000, Campbell Scientific^®^, Logan , UT, USA), a relay controller (SDM-CD16AC, Campbell Scientific^®^, USA), and a channel multiplexer (AM16/32B, Campbell Scientific^®^, USA). The air velocity inside the wind tunnel was set at three different rates: minimum (0.2 m/s (min)), median (1.7 m/s (med)), and maximum (3.2 m/s (max)). Such speeds were achieved by adjusting the fan power and systematically measuring its speed at eight geometrically distributed points on the sample’s surface using a Type K/J hot wire anemometer (YK-2005AH model, Impac Comercial e Tecnologia Ltda, Vargem Grande Paulista, Brazil)). The focus of this study was on a qualitative and comparative analysis of internal and external temperatures, as well as surface temperatures of the roofing element, under specific conditions. These findings aimed to highlight trends in thermal behavior rather than a precise quantitative modeling. As for the equipment used, it is important to mention the advantages of modern and more advanced tools. However, due to limitations in available resources, accessible instrumentation was utilized, such as thermocouples (type K), thermal cameras, dataloggers, and wind speed controllers. Despite these constraints, the methodologies presented were robust and the data collected were reliable within the scope of the study.

**Figure 4:**
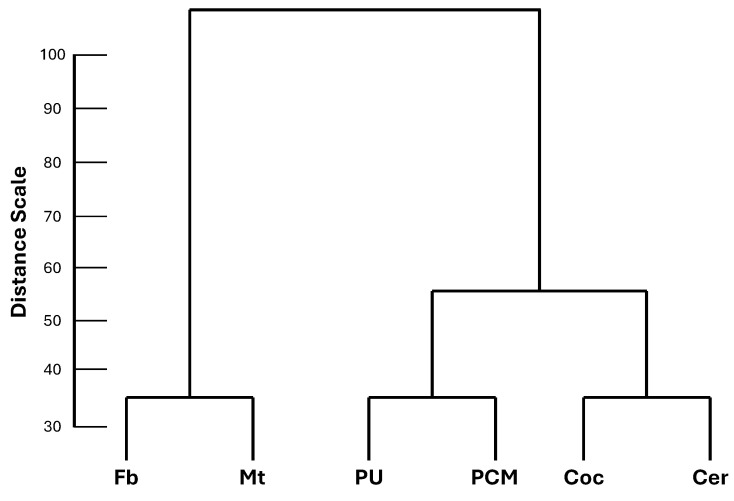
Method of grouping data: dendrogram of internal surface temperature of materials evaluated at median air velocity (1.7 m/s).

The profiles of internal surface temperature at a median air flow speed (1.7 m/s) for each material evaluated over time were analyzed using agglomerative hierarchical cluster analysis (Figure 4), a multivariate method. This technique groups objects into a multilevel cluster tree (dendrogram) base on their similarity [27], interconnecting samples according to their associations [28]. The classification of the objects was carried out using Ward’s agglomerative hierarchical method, and dissimilarity was measured through Euclidean distance [28]. Ward’s linkage method is based on a classical sum-of-squares criterion, which generates groups that minimize within-group dispersion at each binary fusion. The Euclidean distance calculates the straight-line between two points in n-dimensional space, dividing the linkage distance by the maximum distance and multiplying by 100 [27].

## 3. Results and Discussion

### 3.1. Characterization

The impregnation achieved in all samples was greater than 14% in weight. This is substantially higher than what was achieved with the impregnation method using the solid culm without immersion in other bamboo species such as *Phyllostachys aurea* (7.11%) [15]. Impregnation reached an average of 15.16% with PCM and 14.39% with coconut oil with an original average apparent basic density of 0.41 g/cm^3^.

#### 3.1.1. Microscopy and Ultrastructural Analysis

**Figure 5:**
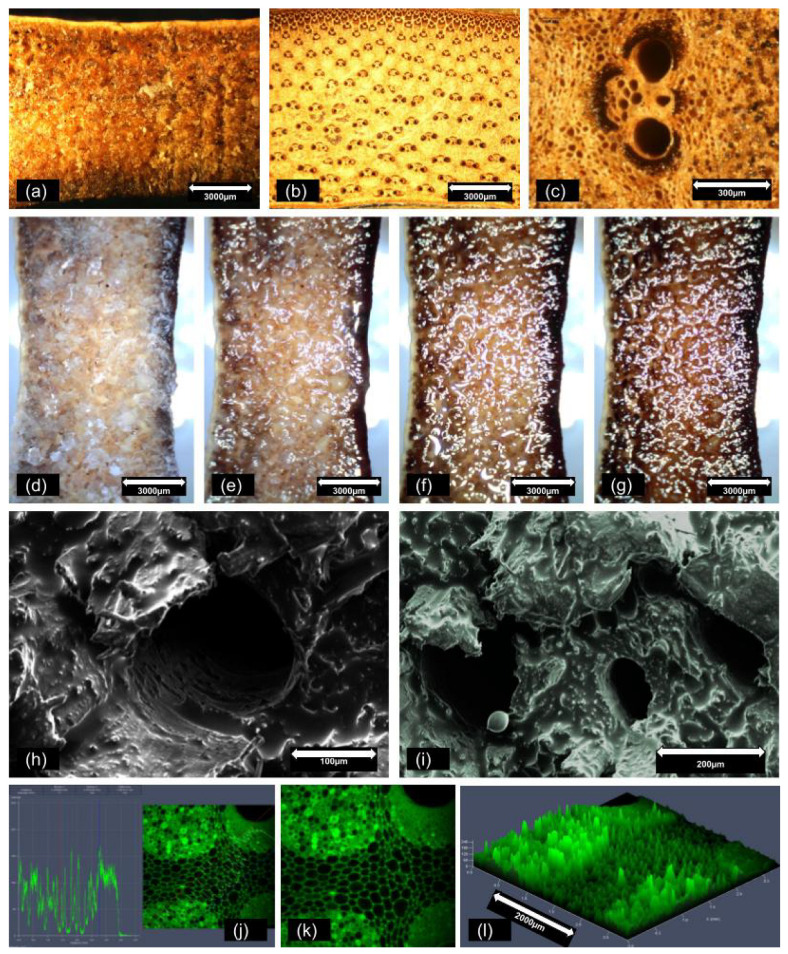
Stereomicroscope images (**a**–**c**) (at 10× (**a**,**b**) and 125× magnification (**c**)) of the internodal cross-sections of PCM-impregnated (**a**) and raw (**b**,**c**) *D. giganteus* bamboo culms; time-lapse images at 10× magnification of the melting of PCM within bamboo culms, taken every 20 s (**d**–**g**); images of the internodal cross-sections (**h**,**i**), obtained by ultra-high-resolution field emission scanning electron microscope (SEM) and confocal microscope (**j**–**l**).

Figure 5a–c show stereomicroscope images of the internodal cross-sections of bamboo samples, in which the porosity of the bamboo species can be seen. Notably, larger voids can be seen within the sap-conducting vessels of the parenchyma, a structure primarily composed of lignin, which forms the cellular framework. In Figure 5a, the sample has been impregnated with PCM, displaying fewer voids and a waxy appearance. Comparatively, Figure 5b,c show bamboo in its natural state, displaying a larger number of voids and distinct sap-conducing vessels.

Figure 5d–g present time-lapse images of bamboo cross-sections containing PCM when exposed to heat. Images were taken every 20 s under 10× magnification. The initially solid PCM begins to melt, potentially leading to leakage in case of the absence of a coating material such as the PU resin.

In Figure 5h,i, in the images obtained by SEM, it is possible to observe, on a comparative scale, the large voids present in the anatomical microstructure of bamboo plant tissues. In Figure 5j–l, we observe the scanning of the cross-sectional surface of the bamboo culms performed using the confocal microscope, with an assessment of its roughness. Both the three-dimensional image and the surface profile of the section demonstrate how rough it is.

#### 3.1.2. Thermogravimetric Analysis (TGA)

**Figure 6:**
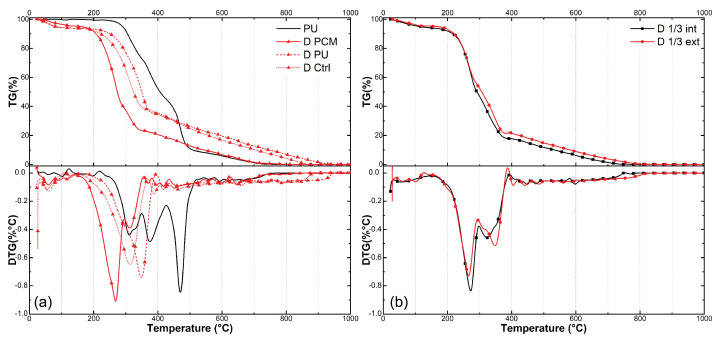
Thermogravimetric analysis curves (TG and DTG) for raw PU resin (PU), PU-coated bamboo (D PU), PCM-impregnated (D PCM), and raw *D. giganteus* bamboo (D Ctrl) samples (**a**); inner (D 1/3 int) and outer (D 1/3 ext) thirds of bamboo walls (**b**).

Figure 6 shows thermogravimetry (TG) and derivative thermogravimetry (DTG) curves of raw PU resin and both raw and treated bamboo samples. The initial mass loss observed up to 100 °C is attributed to the loss of free water in bamboo samples (Figure 6a) [13]. It can be observed that the bamboo sample containing PCM (D PCM) had a lower mass loss at this stage in comparison with raw (D Ctrl) and PU-coated (D PU) bamboo, which can be attributed to the PCM filling voids that could normally be filled by water, as observed in the previous section. Further noteworthy mass loss events can be discerned in these curves, as follows:

In the raw bamboo sample (D Ctrl) DTG curve (Figure 6a), there are peaks associated with the main constituents of bamboo, including hemicelllulose (250–320 °C), cellulose (348 °C), and lignin (250–600 °C) [29]. These peaks are also found in treated bamboo samples.

In the PCM-treated (D PCM) sample curve (Figure 6a), a peak emerges shortly after 200 °C, distinguishing it from samples treated without PCM. This disparity arises from the degradation of PCM, an organic ester, commencing at relatively low temperatures [30].

According to [31,32] , polyurethane (PU) experiences mass loss in two decomposition stages: the rupture of urethane bonds between 240 °C and 350 °C, and the decomposition of ester bonds within the polyol at 500 °C. These peaks are distinguishable in PU-coated bamboo samples (D PU) (Figure 6a), although there is some overlap with the bamboo constituents’ degradation occurring at similar temperatures.

The samples depicted in Figure 6b were included in the analysis to highlight visible differences in the impregnation of the anatomical tissues within the walls of the bamboo culm internodes. There is a notable variation in vascularity and, consequently, porosity between the innermost parts of bamboo walls and those closer to the outer limits. In this case, samples were specifically taken from the inner third and the outer third, excluding the intermediate third. The most prominent peak is situated between 200 °C and 300 °C, with the subsequent peak, which typically corresponds to plant tissue constituents in other samples, being diminished. These observed variations indicate changes in the proportion of material components in these two distinct layers. However, only a subtle difference is noted in the degradation temperature of the PCM, which does not clearly reveal variations in the quantity of this substance.

#### 3.1.3. Fourier Transform Infrared Spectroscopy (FTIR)

**Figure 7:**
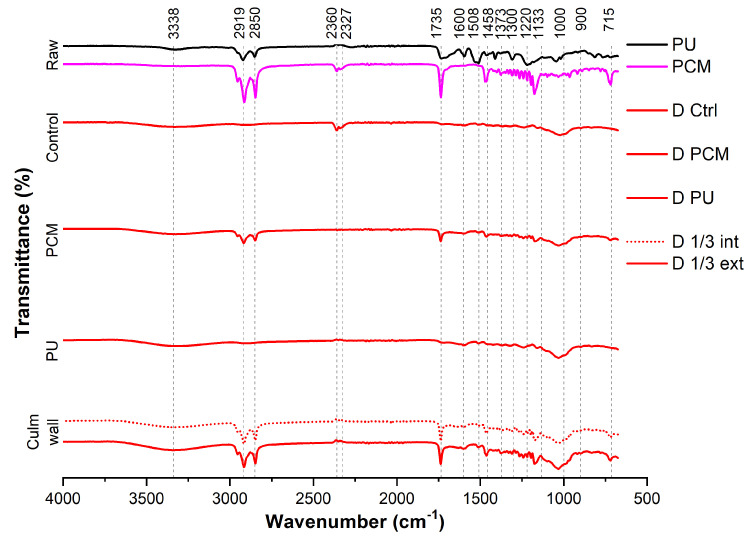
FTIR spectroscopy of PU, PCM, raw bamboo (D Ctrl), PU-coated bamboo (D PU), and PCM-impregnated bamboo (D PCM); comparison between inner and the outer third of bamboo walls samples containing PCM (D 1/3 int and D 1/3 ext).

Fourier Transform Infrared Spectroscopy (FTIR) was utilized to identify functional groups primarily in plant fibers [33], as well as chemical bonds in materials. The samples used were the same as those in the TG analysis, in addition to raw PCM. The results are derived from the absorption of radiation at various wavelengths, spanning from 4000 to 500 cm^−1^ [34] , as depicted in Figure 7. Notably, the active peaks observed in all bamboo samples exhibit a striking degree of similarity, suggesting minor variations in intensity. The existing literature suggests that each absorbance band is associated with characteristic chemical functional groups in specific materials, such as lignocellulosic substances [35].

Bamboo primarily consists of lignin, cellulose, hemicellulose, starch, silica, and pectin. According to [36], the bands between 1600 and 1450 cm^−1^ represent the characteristic region of lignin, hemicellulose, cellulose, and pectin, with peaks between 1558 and 1508 cm^−1^ indicating the presence of lignin, as well as peaks between 1338 and 1234 cm^−1^.

Two peaks at 2919 and 2850 cm^−1^ are related to the stretching of C–H bonds. These bonds are found in PU and especially in PCM structures, resulting in an increase in the intensity of these peaks in treated samples. A sharp peak at 1735 cm^−1^, related to the stretching of C=O bonds, is present in the PCM spectrum. This peak can be observed in treated bamboo samples containing PCM.

These results help to confirm that no significant chemical reactions occur during the impregnation process. Additionally, they help to verify the presence of PCM in treated bamboo samples, adding to the confirmation that the vacuum-assisted method of PCM impregnation was successful.

### 3.2. Thermal Variation in Wind Tunnel Assay

The internal surface temperature profiles at a median air flow speed (1.7 m/s) for six roofing materials subjected to dynamic heat transfer in a climate-controlled wind tunnel were used to evaluate their thermal behavior. The dendrogram (Figure 4), a method of grouping data, shows the level of similarity and the number of groups formed on the vertical and horizontal scales, respectively. It is noteworthy that the internal surface temperature profiles can be divided into two groups. The first group (composed by Fb and Mt) and the second group (consisting of PU, PCM, Coc, and Cer) exhibit similar profiles within each group and differ from one another. The second group can be divided into two subgroups, one composed of PU and PCM, and the other composed of Coc and Cer. It is important to note that the entire dataset, spanning the 240 min duration of each test, was used to perform the agglomerative clustering analysis. This likely influenced the number of groups formed, as some of the data points may not have been affected by the PCM and coconut oil impregnation during the heating and cooling phases. Even so, the superior performance of the materials in the second group is evident, as detailed bellow.

**Figure 8:**
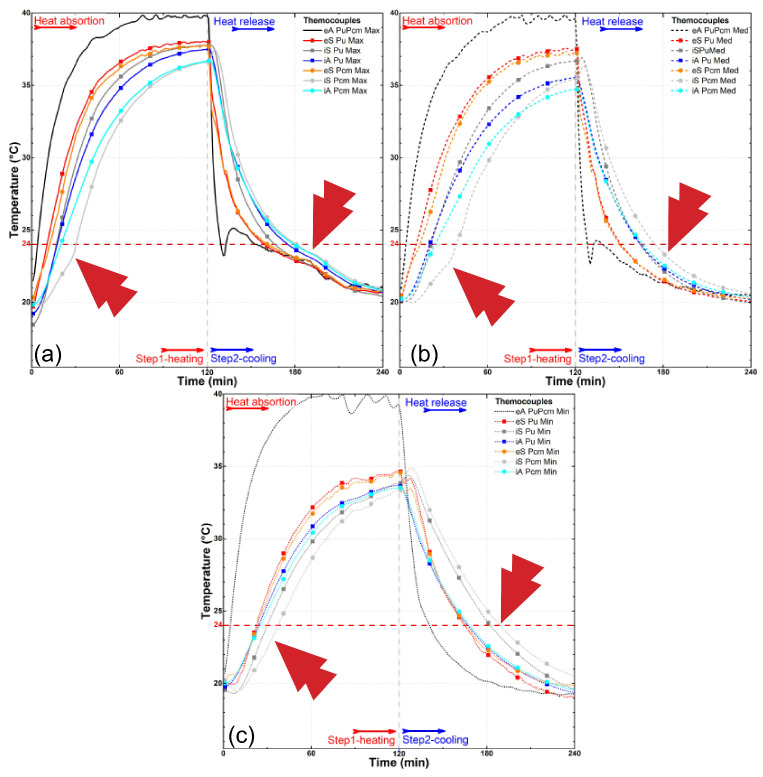
Temperature profiles for the thermometers at the wind tunnel experiment for coated bamboo with polyurethane resin (PU) and PCM-impregnated bamboo (PCM) at maximum (**a**), median (**b**), and minimum (**c**) air flow speed. The arrows highlight points of special interest.

**Figure 9:**
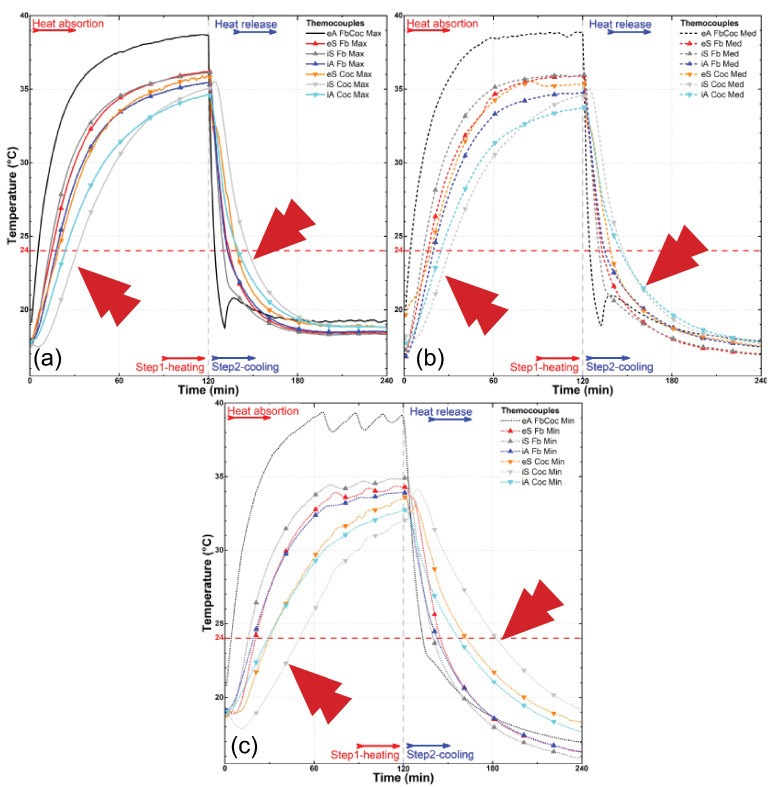
Temperature profiles for the thermometers at the wind tunnel experiment for coconut oil-impregnated bamboo (Coc) and fiber cement tile (Fb) at maximum (**a**), median (**b**), and minimum (**c**) air flow speed. The arrows highlight points of special interest.

**Figure 10:**
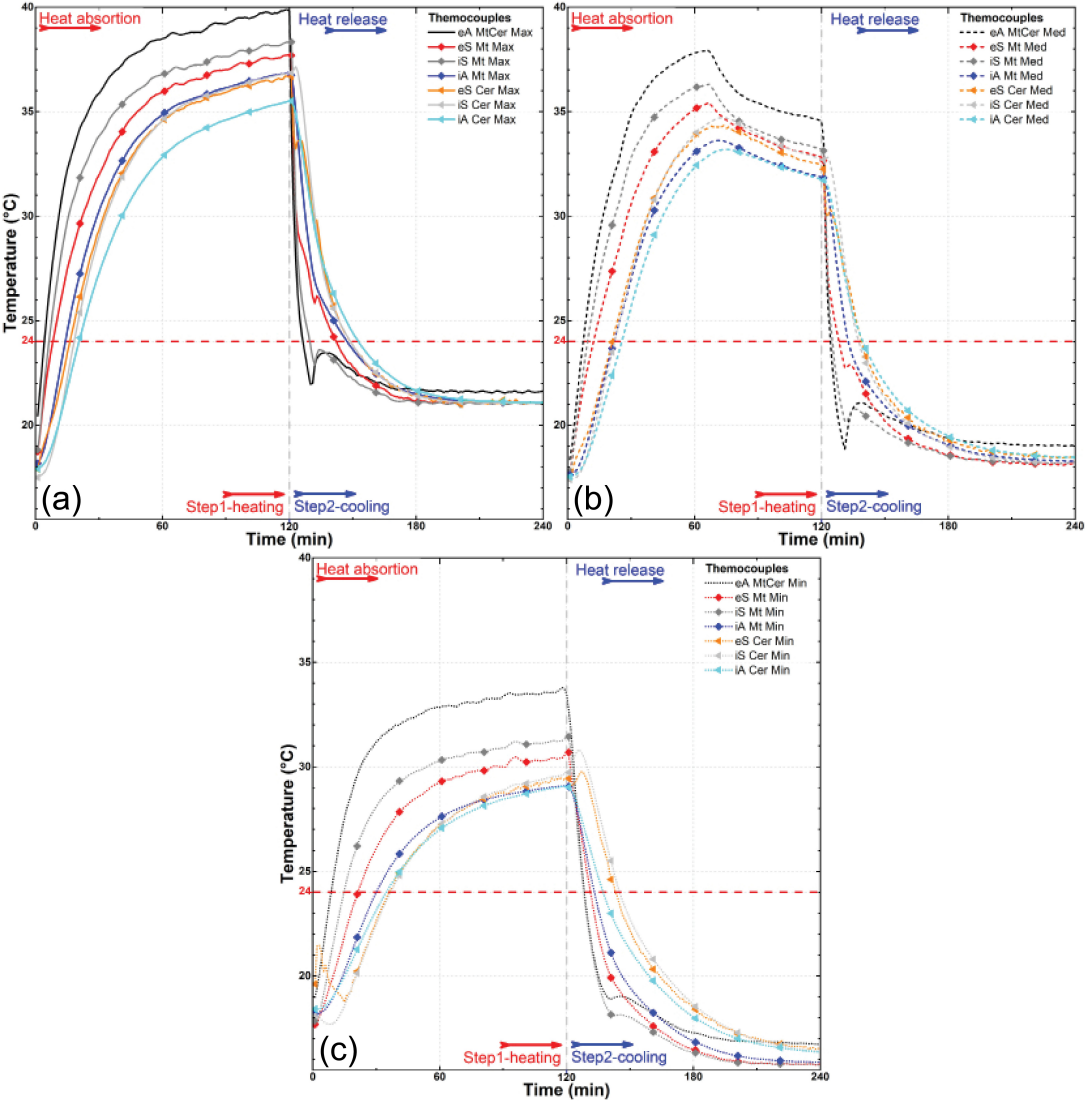
Temperature profiles for the thermometers at the wind tunnel experiment for ceramic roof tile (Cer) and metal plate (Mt) at maximum (**a**), median (**b**), and minimum (**c**) air flow speed.

**Figure 11:**
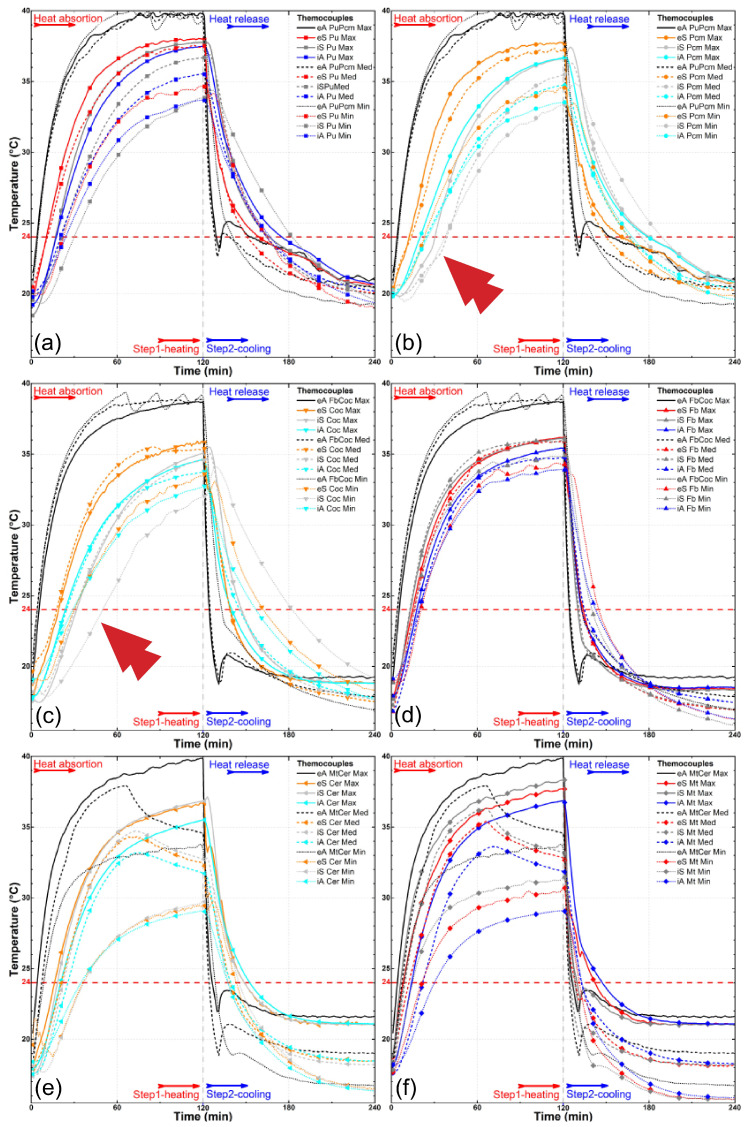
Temperature profiles for the thermometers at the wind tunnel experiment for the three different wind speeds (max, med, min) in each sample: (**a**) PU-coated bamboo, (**b**) PCM-impregnated bamboo, (**c**) coconut oil-impregnated bamboo, (**d**) fiber cement tile, (**e**) ceramic roof tile, and (**f**) metal plate. The arrows highlight points of special interest.

**Figure 12:**
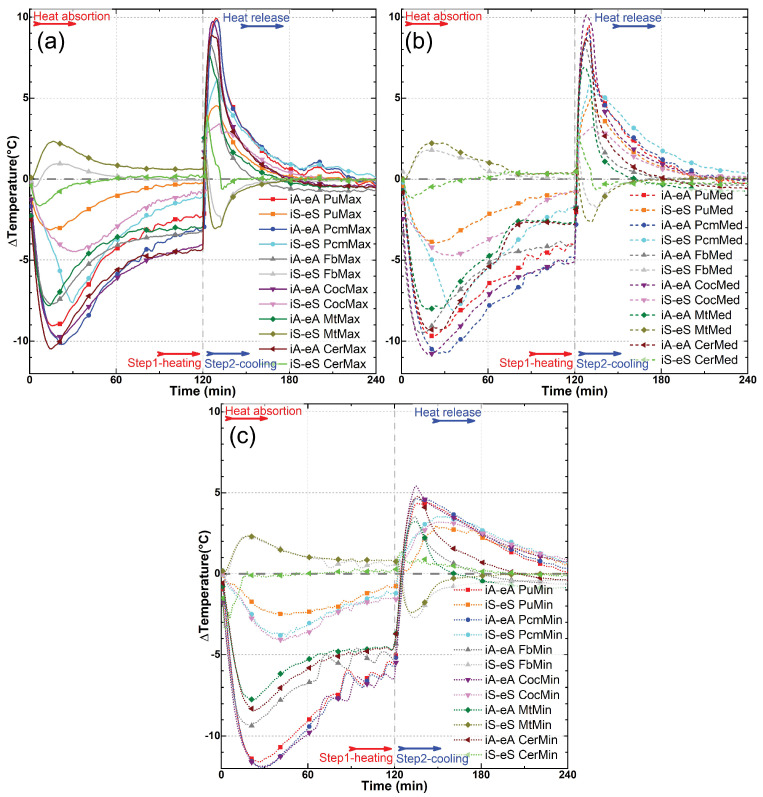
Temperature profiles for differences between the external air minus the internal air (iA−eA), and the external surface minus the internal surface (iS−eS) of all the samples at maximum (**a**), median (**b**), and minimum (**c**) air flow speed.

**Figure 13:**
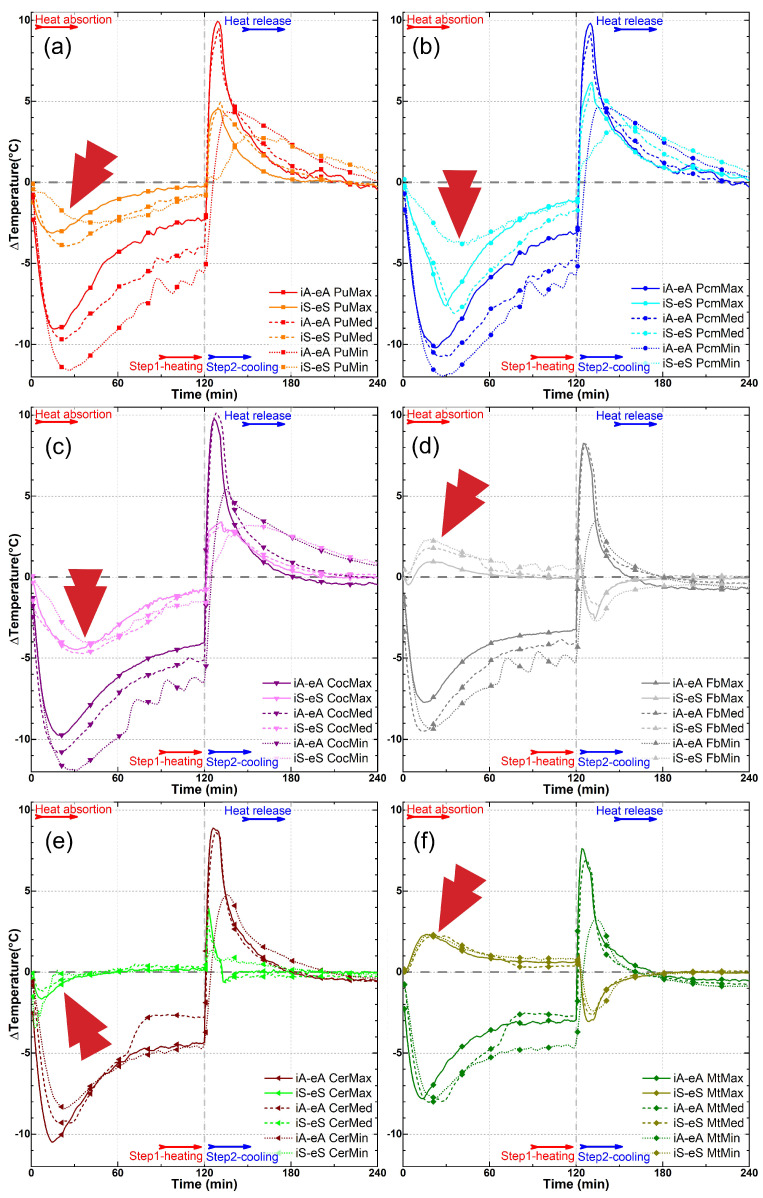
Temperature profiles for differences between the external air minus the internal air (iA−eA), and the external surface minus the internal surface (iS−eS) in each isolated sample: (**a**) PU-coated bamboo, (**b**) PCM-impregnated bamboo, (**c**) coconut oil-impregnated bamboo, (**d**) fiber cement tile, (**e**) ceramic roof tile, and (**f**) metal plate. The red arrows indicate the ranges where the peaks of difference between temperatures occur in the heating phase of the setup, facilitating their identification.

The results of the experiment are depicted in Figure 8, Figure 9, Figure 10, Figure 11, Figure 12 and Figure 13, illustrating temperature profiles recorded by the thermometers positioned within the setup, as previously detailed, as well as the subtraction between the antagonistic positions of the present thermometers. The samples underwent thermal testing, encompassing both heating and cooling cycles (Phases 1 and 2).

The results were presented individually for each specimen in the proposed treatments (Figure 8, Figure 9 and Figure 10) and combined for better data comparison visualization (Figure 11). The solid, dashed, and dotted lines represent, respectively, the maximum (3.2 m/s), median (1.7 m/s), and minimum (0.2 m/s) air flow speed. Each color represents the average measurement of a pair of thermometers positioned simultaneously in the regions as described, with eA, eS, iS and iA, respectively, in black, red/orange, gray/light gray, and blue/cyan colors, taking into account the different types of samples; it is also possible to distinguish each specimen by its specific symbol as shown in the lines.

The results presented in Figure 8 show that the temperature variation profile in the thermometers of the PCM samples and those resin-coated with PU are quite distinct despite a similarity, in both samples, with the temperatures of the external air and the external surface of the samples rising rapidly due to the fast heat increase at the wind tunnel environment (Figure 8a–c). However, there is a significant delay in heating the samples’ internal surfaces and an even more prominent delay in the interior air of the passive chamber in the same samples. This delay is, however, much more pronounced when we look at the curve of the sample treated with PCM, highlighting that even the external surface of the PCM-treated sample experiences a slight delay in temperature increase.

A comparison between the PU and PCM samples shows that the temperature of the resin-coated bamboo was slightly higher in all thermometers; however, there is no visual evidence of a change in the temperature growth trend, as the curves exhibit a constant linear trend, with parallelism between the external air (eA) and all the other thermometers. However, when the PCM-impregnated sample reaches approximately 21–24 °C, the curve trends begin to shift. This shift coincides with the PCM undergoing a phase change, absorbing heat, and consequently delaying the temperature increase. For instance, while the internal surface thermometers of the non-impregnated samples (iS PU) take approximately between 15 and 20 min to reach 25 °C (crossing the melting point of the PCM), the PCM-impregnated sample (iS PCM) requires approximately up to 40 min, indicating a relevant longer time to reach the same temperature. This result demonstrates the efficacy of PCM-impregnated bamboo culms for thermal energy storage where there is visibly an action by the PCM in anchoring the temperature change in physical state around 24 °C.

Another clearly visible aspect in these three illustrated graphs (Figure 9a–c) is the difference in the speed at which all thermometers, with the exception of the external air (eA) in the wind tunnel environment, exhibit a slowing down of their heating as the wind speed decreases, demonstrating a significant influence of the air flow velocity on the heat exchange occurring in the system. While the external environment, for example, exceeds 35 °C in all three speeds tested in about 10 min of the experiment (eA PuPcmMax, Med, or Min, Figure 9a–c), with a maximum airflow speed of 3.2 m/s, the resin-coated samples (eS PuMax, Figure 6a) reach this threshold only after approximately 40 min at medium speed (1.7 m/s), and after about 55 min at the minimum speed of 0.2 m/s, they do not even reach this 35 °C level.

There is also an unexpected observable effect in the three presented graphs (Figure 8). The temperatures of the internal surfaces (iS PcmMax, Med, and Min) of the PCM-containing samples exhibit a greater delay in their heating and cooling processes than the internal air (iA PcmMax, Med, and Min) of the supposedly insulated passive chamber. This effect is likely related to the gaps present in the composition of the sample units in the partition made. In other words, despite the delay caused by the PCM’s temperature anchoring and heat accumulation effects, due to the existence of spaces for airflow and the added wind speed, this partition of the samples does not prove to be effective or sufficient for retaining the passage of this energy.

When it comes to the secondary samples in this research, which serve the purpose of comparison with conventional building materials as well as coconut oil-impregnated bamboo in comparison with the PCM’s performance, we can observe in Figure 9 and Figure 10, with regard to the latter (Figure 9), that the thermal behavior revealed in the displayed curves is highly similar to the behavior observed with PCM impregnation. There is a significant delay in temperature gain, with the internal air of the passive chamber (iA Coc) reaching higher temperatures than the internal surface (iS) of the samples, once again revealing the possible influence of gaps. However, there is no significant distortion in the curve’s trend, as observed in the case of the commercial PCM. In other words, there is no clear anchoring to a specific temperature range; instead, there is a spreading of this effect.

The effect of wind speed (Figure 9 and Figure 10) on the rate of heat exchange is also noticeable in all samples, as observed in Figure 8, further emphasizing this influence. As for the behavior of the three conventional materials analyzed—fiber cement tiles, metal plates, and ceramic tiles—the results show similar behavior among them. There is a parallel trend between the variation in external air temperature (eA) and all other variations, both in the external surfaces (eS) and internal surfaces (iS) of the samples, as well as in the internal air (iA) of the passive chamber. In these cases, the internal air consistently showed a delayed calorie gain and loss compared to the overall system, unlike what was observed in the bamboo samples.

The data presented in Figure 11, which compile temperature profiles for each sample at the three wind speeds, clearly illustrate the individual effect of airflow velocity on temperature gains at various points in the setup. For instance, in Figure 11b,c—showing the PCM and coconut oil-impregnated samples, respectively—the separation between the dotted lines (indicating the lowest wind speed), dashed lines (median speed), and solid lines (maximum speed) illustrates the significant impact of airflow on temperature distribution. Additionally, the curves representing minimum speed exhibit more pronounced roughness, indicating irregularities in the heat exchange process at lower velocities. This behavior contrasts with the smoother curves observed at higher air velocities.

The results that can be observed in Figure 12 and Figure 13 also demonstrate the analyses conducted regarding the temperature difference (ΔT) between the profiles measured for the external air as opposed to the internal air (iA−eA); additionally, the differences between the external surface and the internal surface (iS−eS) highlight the delay in conduction and the storage of thermal energy through the samples.

A greater difference between the internal air and the external air is noticeable in almost all cases, as well as the tendency for the difference to stabilize when the variation in the temperature at the wind tunnel environment also stabilizes, which is always around the 90 min. A larger difference is also evident in the PCM, coconut oil, and PU samples, in that order, both in terms of the difference between the internal and external surfaces and between the internal and external air (Figure 13a–c).

Regarding the samples treated with PCM (Figure 13b), there is also a clear distortion in the graph profile with its maximum point occurring around the 30th minute of the experiment, where a peak can be observed. This peak is also noticeable during the second phase of the experiment, where the external air temperature becomes lower than the internal air temperature after the 180th minute. These peaks are likely caused by the action of the PCM, which anchors the temperature due to the storage of thermal energy, either absorbing or releasing heat. This effect is also not observed in the samples with coconut oil (Figure 13c).

When observing the samples of conventional building materials (Figure 13d–f), it is possible to notice a reversed effect in the temperature gain expressed on the surfaces of the samples (iS-eS) in both the metal plate and fiber cement samples (Figure 13d,f). The properties of high thermal conductivity of these materials are widely known, and this effect is not observed in the ceramic tile (Figure 13e), which is also known for being a more effective thermal insulator. Nevertheless, when comparing ceramic with all bamboo samples, whether impregnated or not, substantial differences are observed.

## 4. Conclusions

This study represents a significant advancement in the exploration of processes that can facilitate the utilization of bamboo as a support material for PCM impregnation. In this research, bamboo culms are impregnated under negative pressure while maintaining their typical practical shape and sealed with polyurethane resin to create a thermally efficient composite material. The thermal variation wind tunnel analysis highlights the material’s ability to stabilize temperatures around the specific melting point of the PCM (24 °C), underscoring its functionality. However, it also reveals that the current method of sample preparation may not fully maximize this capability.

The presence of increasing airflow demonstrates the samples’ capacity to store thermal energy, but they are unable to entirely prevent this energy from transferring into the passive chamber used in the primary experiment, mainly because of the gaps contained in the samples. Nonetheless, it also indicates that heat exchange occurs in direct proportion to the wind speed.

Comparative studies involving conventionally employed materials in civil construction strongly emphasize the distinct behavior of PCM. Nevertheless, the comparison made with bamboo impregnated with coconut oil does not reveal a significant improvement in thermal efficiency that would qualify this alternative material for similar use as commercially manufactured industrial PCMs.

Furthermore, FTIR, TG, and DTG analyses indicate that no notable chemical reactions take place during the impregnation process, which is advantageous for its application in phase change thermal energy storage. This process ensures the efficient penetration of the PCM into the porous structure of the bamboo culms. Consequently, the developed composite material holds promise as a versatile bamboo-based composite that serves multiple functions and has the potential for applications related to energy-saving in building thermal conditioning. It is anticipated to enhance the energy efficiency of buildings and other related purposes.

The results of this study also highlight the importance of conducting more comprehensive analyses regarding the possibilities of impregnating bamboo culms with PCMs. Other bamboo species with specific characteristics may offer more intriguing capabilities for this application. Additionally, different impregnation methods and thermal analysis techniques should be explored to assess potential process improvements.

## Figures and Tables

**Table 1:** Physical properties of CrodaTherm 24.

Property	Typical Value	Unit
Thermal properties by differential scanning calorimetry (DSC)		
Peak melting temperature	24	°C
Latent heat (melting)	184	kJ/kg
Peak crystallization temperature	22	°C
Latent heat (crystallization)	−182	kJ/kg
Thermal properties by three-layer calorimetry (3LC)		
Peak melting temperature	23	°C
Total heat capacity (melting)	207	kJ/kg
Peak crystallization temperature	23	°C
Total heat capacity (crystallization)	213	kJ/kg
Other properties		
Bio-based content	100	%
Density at 20 °C (solid)	906	kg/m³
Density at 40 °C (liquid)	843	kg/m³
Flash point	226	°C
Specific heat capacity (solid)	3.7	kJ/kg °C
Specific heat capacity (liquid)	2.2	kJ/kg °C
Volume expansion 20–40 °C	7.5	%
Thermal conductivity (solid)	0.22	W/m °C
Thermal conductivity (liquid)	0.16	W/m °C
Thermal cycles without change in properties	10,000	cicles

**Table 2:** Treatmentand labels of the samples used in the thermal variation in wind tunnel assay.

Treatment	Objective	Label
Bamboo specimens (*Dendrocalamus giganteus*)		
Sealed with PU	control	PU
PCM impregnated and sealed with PU	analysis 1	PCM
Coconut oil impregnated and sealed with PU	analysis 2	Coc
Commonly used building materials		
Ceramic tiles	reference 1	Cer
Fiber cement tile	reference 2	Fb
Metal sheet	reference 3	Mt

**Table 3:** Morphological characteristics of the original bamboo samples.

Culms Average General Dimensions (mm)			Average Characteristics of the Nodes	
Diameter [Ø]	Length [l]	Wall thickness [t]	Amount	Average distance (mm) [n]
140	250	8	0	450

**Table 4:** Morphology, organization, and dimensions of the samples as placed at the passive chamber.

Species	Cross-Sectional Diagram and Sample Organization	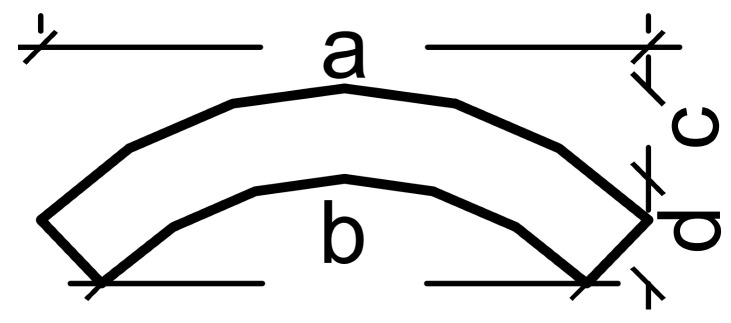
Average dimensions (mm)		
*Dendrocalamus giganteus*	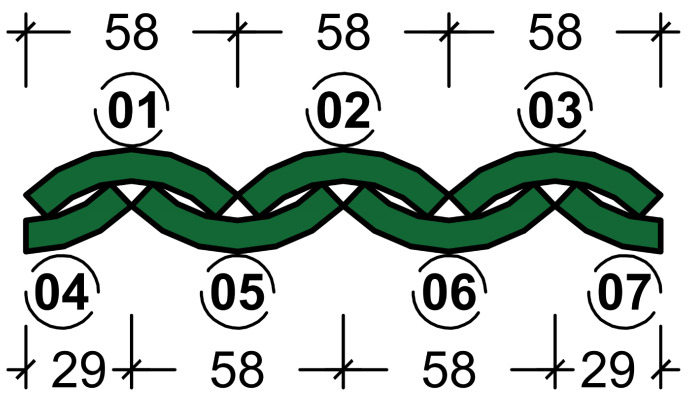	a = 58; b = 50; c = 8; d = 7

## Data Availability

Data that support the findings of this study are available from the corresponding author upon reasonable request (the data are part of ongoing research and are subject to intellectual property considerations. Public disclosure at this stage could compromise potential future publications or commercialization opportunities. Access can be granted upon request to ensure responsible data sharing).
